# An Unusual Presentation of Multifactorial Hypocalcemia as Myopathy: A Case Report

**DOI:** 10.7759/cureus.87434

**Published:** 2025-07-07

**Authors:** Aloke Aloke, Komal Singh

**Affiliations:** 1 Department of Medicine, Tata Main Hospital, Dhanbad, IND; 2 Department of Obstetrics and Gynecology, Tata Main Hospital, Dhanbad, IND

**Keywords:** blood transfusion, idiopathic hypoparathyroidism, myopathy, sepsis, severe hypocalcemia, vitamin-d deficiency

## Abstract

Hypocalcemia is a common metabolic disturbance with a broad spectrum of clinical presentations, ranging from asymptomatic laboratory findings to severe, life-threatening complications. While neuromuscular irritability is a well-known feature, myopathy as a primary presentation is rare and often under-recognized. We report the case of a 45-year-old female with type 2 diabetes and hypertension who presented with progressive lower limb weakness and generalized body aches. She had a recent history of an abdominal skin abscess and had received blood transfusions without calcium correction. On examination, she demonstrated significant proximal muscle weakness and elevated serum creatine kinase levels. Laboratory investigations revealed severe hypocalcemia (serum calcium 4.5 mg/dL), low vitamin D, and inappropriately low-normal parathyroid hormone (PTH) levels. ECG showed QTc prolongation and anteroseptal injury pattern. A diagnosis of hypocalcemic myopathy secondary to idiopathic hypoparathyroidism, compounded by vitamin D deficiency, sepsis, and transfusion-related citrate load, was made.

The patient was treated with intravenous calcium gluconate, vitamin D supplementation, and antibiotics. Her muscle strength gradually improved with normalization of calcium levels. She was discharged on oral calcium and calcitriol, and showed near-complete recovery at the two-week follow-up. This report highlights the importance of considering hypocalcemia in patients presenting with myopathy. Multiple contributing factors can coexist and exacerbate calcium deficiency in these patients. Early recognition and prompt correction are essential to prevent long-term neuromuscular complications.

## Introduction

The presentation of hypocalcemia can range from an incidental laboratory finding to a severe, life-threatening condition, depending on both the degree of calcium deficiency and the rapidity of onset. [1] Among the most common etiologies are impaired parathyroid hormone (PTH) secretion and deficient vitamin D synthesis. [2] Additional causes include low magnesium, sepsis or other critical illnesses, certain medications such as phenytoin and bisphosphonates, as well as PTH resistance syndromes. Clinically, hypocalcemia often manifests with symptoms such as paresthesia, seizures, carpopedal spasm, bronchospasm, and a prolonged QT interval on ECG. However, myopathy as a primary and only presenting feature is an uncommon and under-recognized manifestation [3].

We present the case of a female patient with a known history of diabetes mellitus and hypertension for the past four years. Her blood sugar and blood pressure levels were well controlled with medication. Over a span of two weeks, she had developed a superficial abdominal abscess and had been treated at a local nursing home. As part of her treatment, she had received antibiotics and undergone a transfusion of three units of blood. While investigations had revealed hypocalcemia during her stay, it had not been addressed. Following discharge, the patient developed proximal muscle weakness and was subsequently admitted to our hospital. On evaluation, she was found to have severe hypocalcemia, which manifested predominantly as proximal myopathy. Management focused on correcting the hypocalcemia, and further investigations were carried out to identify the underlying causes. It was determined that there were two primary contributors to her hypocalcemia: hypoparathyroidism and vitamin D deficiency. In addition, the recent sepsis and blood transfusion may have further aggravated the hypocalcemic state. This case is notable for the presence of multiple contributing factors leading to severe hypocalcemia in the patient, which in turn resulted in proximal myopathy.

## Case presentation

A 45-year-old female with a history of type 2 diabetes mellitus and hypertension, well-controlled on regular medications for the past four years, presented to the outpatient department in a wheelchair with complaints of generalized body aches and progressive weakness of both lower limbs for the past three to four days. Fifteen days before presentation, she had developed a skin abscess over the left upper abdomen, for which she had been prescribed oral antibiotics by a local practitioner. During subsequent investigations at a local nursing home, she had been found to have anemia and hypocalcemia. She had been admitted and received three units of blood transfusion and other supportive treatments. However, her hypocalcemia had not been addressed, and she had been discharged without calcium correction. 

**Figure 1:**
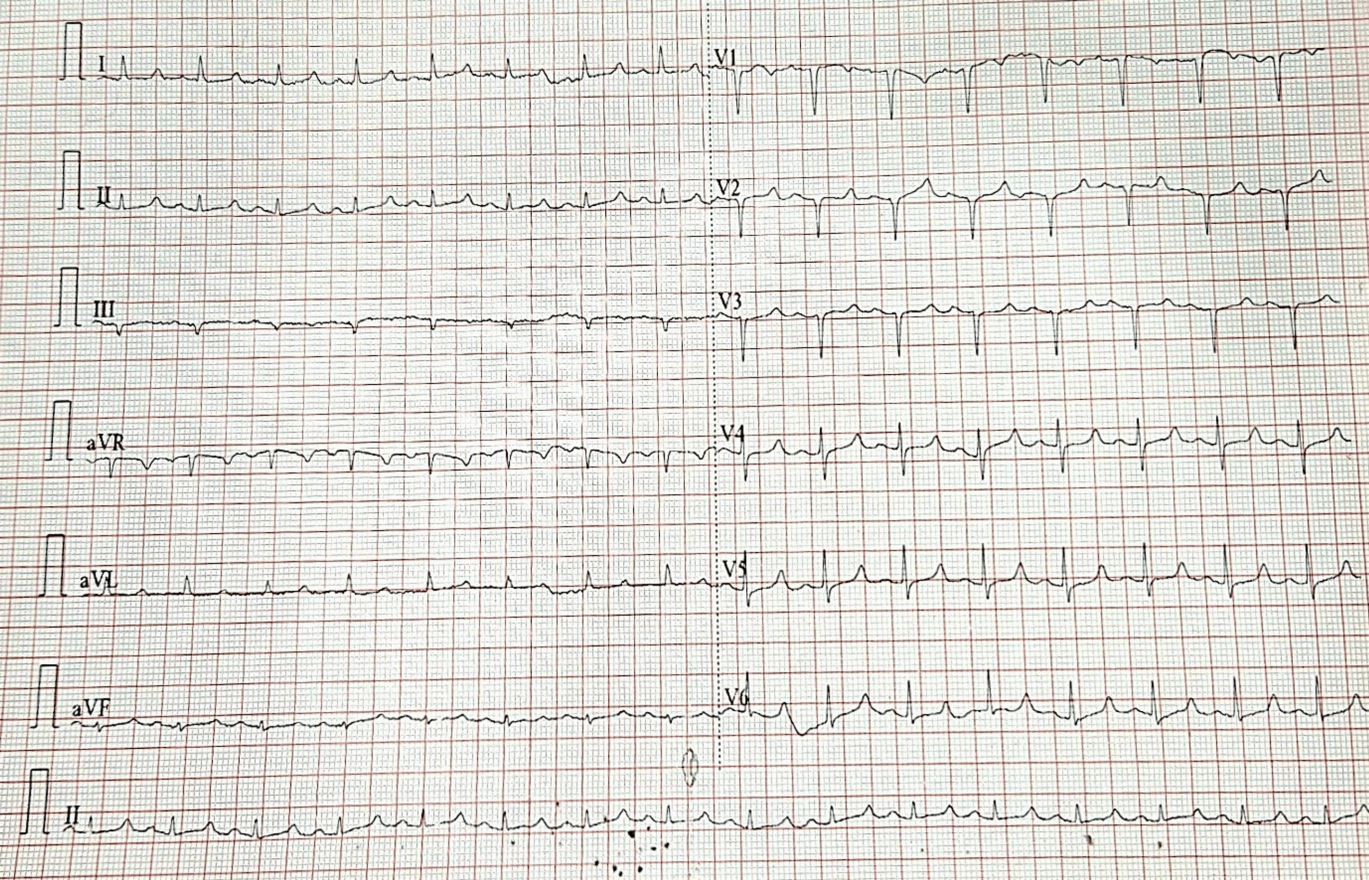
ECG of the patient A 12-lead electrogram showing QTc interval of 486 ms on ECG (suggestive of borderline prolongation) and pattern of acute anteroseptal injury ECG: electrocardiogram

Her regular medications included glimepiride 2 mg, metformin 1 gram, and telmisartan 40 mg daily for management of diabetes and hypertension. Following discharge, she had begun experiencing worsening body aches and weakness, particularly in the lower limbs, eventually becoming bed-bound and unable to walk. The weakness was progressive and symmetrical, more pronounced in the lower limbs but also involving the upper limbs. She denied any bowel or bladder involvement, back ache, history of trauma, surgery, alopecia, cutaneous changes, or cataracts. She reported a remote history of prolonged fatigue and reduced exercise tolerance. On examination, the patient was obese and appeared pale. Pulse and blood pressure were stable. She was alert and oriented, with intact higher mental functions. Cardiovascular and respiratory system examinations were unremarkable. Neurologically, she had flaccid muscle tone with absent deep tendon reflexes in the lower limbs. Muscle strength was graded as 3+/5 in the upper limbs and 1/5 in the lower limbs bilaterally. No fasciculations or sensory deficits were noted. Chvostek’s and Trousseau’s signs were negative. Diagnosis of myopathy was made, and investigations were sent. The ECG of the patient is presented in Figure 1.

Investigations (Table 1) revealed severe hypocalcemia with inappropriately low PTH, favoring the diagnosis of hypoparathyroidism along with vitamin D deficiency and high creatinine phosphokinase. Laboratory evaluations revealed normal electrolyte levels, including potassium and phosphorus, and urinalysis showed no myoglobin, effectively ruling out rhabdomyolysis. The patient was diagnosed with hypocalcemic myopathy secondary to hypoparathyroidism and vitamin D deficiency. Other factors could have contributed to hypocalcemia as well, like sepsis and blood transfusion. Treatment was initiated with 10 ml of 10% calcium gluconate IV over 10 minutes, followed by continuous infusion of calcium gluconate (10 ampoules in 1 liter of normal saline at 100 ml/hour) along with intravenous antibiotics and vitamin D supplementation. However, medicines for diabetes and blood pressure were continued. Serum calcium was monitored frequently (every six hours). Over the following hours, when serum calcium levels started improving, there was a gradual improvement in muscle strength. Infusion rate was adjusted to maintain serum calcium at 7-8.5 mg/dL. After five days, when the patient was able to walk without support and blood investigations were normal, she was discharged on oral calcium carbonate (1 g per day), calcitriol (0.25 mcg per day), along with oral antibiotics, antihypertensives, and anti-diabetics. At follow-up after two weeks, the patient had regained near-complete muscle function. She was advised to continue treatment and to be on regular follow-up for monitoring of blood and urinary calcium.

**Table 1:** Summary of investigations performed outside and at our hospital CPK: creatinine phosphokinase; CRP: C-reactive protein; Hb: hemoglobin; LFT: liver function test; PTH: parathyroid hormone; TLC: total leucocyte count; TSH: thyroid-stimulating hormone

Parameters	Reference range	17/4/24 (investigations done outside)	26/4/24 (day of admission)	27/4/24 (in hospital)	28/4/24 (in hospital)	29/4/24 (in hospital)	30/4/24 (at discharge)
Hb, g/dL	12–16	7.5	10.2				
TLC, /mm^3^	5000–10,000	14,000	22,000		13,000	12,000	9,000
LFT		Normal	Normal				Normal
Serum albumin, g/dL	3.5–5.5	3.65	3.63				
Serum calcium, mg/dl	9–10.5	5.61	4.5	7.2	7.6	7.9	9.2
Serum phosphate, mg/dL	3.0–4.5		3.44				
Serum sodium, mEq/L	136–145	Within normal range	Within normal range				
Serum potassium, mEq/L	3.5–5.0	Within normal range	Within normal range				
Serum magnesium, mg/dL	1.60–2.60		2.34				
TSH, mIU/L	0.3–5.0		3.4				
Vitamin D, nmol/L	75–250		30				
Serum creatinine, mg/dL	0.8–1.3		1.2				
PTH, pg/ml	15–65		29.7				
CRP, mg/L	Less than 6		14.52				4
CPK, units/L	10–120		1,272				63.54

## Discussion

Calcium is an essential ion that plays a vital role in numerous physiological functions, including bone health, muscle contraction, nerve transmission, and blood clotting. Normal serum calcium levels range from 8.5 to 10.5 mg/dL. Levels below 8.5 mg/dL are classified as hypocalcemia. The regulation of calcium in the body is a complex process involving several key factors: PTH, 1,25-dihydroxy vitamin D, fibroblast growth factor 23 (FGF23), calcitonin, calcium-sensing receptors (CaSR), and the interplay between serum calcium and phosphorus levels [4,5]. Hypocalcemia can present either as an asymptomatic laboratory finding or as a potentially life-threatening condition [1,6]. It is crucial to differentiate between acute and chronic hypocalcemia and to assess whether the condition is symptomatic or asymptomatic, as this distinction guides appropriate treatment strategies.

Acute hypocalcemia often presents with features of neuromuscular irritability, such as numbness and tingling in the fingers and toes, paresthesia of the extremities, muscle cramps and weakness, fatigue and anxiety, and carpal spasm or tetany. Clinical signs of neuromuscular irritability include Chvostek’s sign and Trousseau’s sign. In addition, cardiac manifestations are notable in acute cases. The most common ECG finding is prolongation of the QT interval. In some cases, ECG may mimic an acute anteroseptal myocardial infarction, showing features such as QS complexes in leads V1 to V3 - a pattern that has been documented in hypocalcemia. Chronic hypocalcemia, on the other hand, may present with more insidious symptoms. Neurological complications may manifest as pyramidal signs due to basal ganglia calcification, personality disturbances. and other non-specific dermatological manifestations, dental abnormalities, ocular findings, such as subcapsular cataracts, or smooth muscle dysfunction in the form of abdominal pain, biliary colic, and dysphagia.

Our patient exhibited isolated muscular weakness, without the classical signs and symptoms of hypocalcemia. However, a significant finding was the prolongation of the QT interval on ECG, along with QS complexes in leads V1 to V3, indicative of an anteroseptal injury pattern (Figure 1). This ECG presentation, although rare, has been associated with hypocalcemia in the literature [7,8]. In patients with hypocalcemia, it is essential to evaluate serum albumin, phosphorus, magnesium, 25(OH) vitamin D, and particularly PTH levels. Of these, the measurement of PTH is key to determining the etiology of hypocalcemia. A suppressed or inappropriately low PTH level in the presence of hypocalcemia confirms hypoparathyroidism as the underlying cause [9]. Our patient had a low-normal PTH level despite significant hypocalcemia, indicating an inadequate parathyroid response. This finding supported a diagnosis of hypoparathyroidism-induced hypocalcemic myopathy. The exact cause of hypoparathyroidism could not be determined. There was no history of neck surgery, radiation exposure, or medication use (such as immune checkpoint inhibitors or anticonvulsants) that could account for reduced PTH secretion. Autoimmune and genetic causes of hypoparathyroidism were deemed less likely in this case, as there were no clinical features suggesting multisystem involvement. Moreover, autoantibodies against parathyroid, thyroid, and adrenal tissues, and GATA3 gene mutation could not be done due to resource limitations. The late age of onset and lack of syndromic features or family history made a genetic or congenital form of hypoparathyroidism unlikely. Laboratory serum magnesium levels were within the normal range, ruling out hypomagnesemia as a contributing factor.

Based on these findings, we diagnosed the patient with idiopathic hypoparathyroidism. Additionally, the patient had low vitamin D levels, which may have compounded the degree of hypocalcemia. A concurrent abdominal abscess for which antibiotics were administered, along with a recent history of three blood transfusions, suggested that sepsis and citrate load from transfusions may have further contributed to the hypocalcemia. Hypoparathyroidism and vitamin D deficiency are well-established causes of hypocalcemia. Additionally, sepsis and multiple blood transfusions are known to contribute to hypocalcemia through various mechanisms, such as impaired PTH secretion, altered calcium homeostasis, and citrate toxicity. However, in this case, the exact degree to which each factor contributed to the patient’s severe hypocalcemia could not be quantified. Nonetheless, the cumulative effect of these overlapping etiologies likely led to the profound biochemical disturbance observed. This combination of multiple contributing factors to hypocalcemia is uncommon and highlights the importance of a comprehensive diagnostic approach in such presentations.

Our patient presented solely with myopathy, without the classical physical signs typically associated with hypocalcemia. Trousseau’s and Chvostek’s signs were notably absent on examination. Electrocardiography revealed only a slight prolongation of the QT interval, a subtle but relevant indicator of hypocalcemia. Myopathy as a presenting feature of hypocalcemia is uncommon but clinically important. Importantly, statin-induced myopathy was ruled out, as the patient was not on any statin therapy. While the neuromuscular manifestations of calcium deficiency are generally attributed to increased neuronal excitability, chronic hypocalcemia can lead to direct muscle fiber dysfunction, which may manifest as proximal muscle weakness and be associated with elevated serum creatine kinase levels. Recognizing this atypical presentation is crucial, as delayed diagnosis and treatment may lead to further neuromuscular compromise.

## Conclusions

This case was unique due to the rare presentation of myopathy as a manifestation of idiopathic hypoparathyroidism. Additionally, the patient was found to have a vitamin D deficiency, which likely contributed to the hypocalcemia. The clinical scenario was further complicated by the presence of sepsis and a history of three blood transfusions, both of which are known to potentially exacerbate hypocalcemia. Thus, the patient’s hypocalcemia was multifactorial in origin, with contributions from idiopathic hypoparathyroidism, vitamin D deficiency, sepsis, and transfusion-related factors. This combination culminated in severe hypocalcemia, ultimately resulting in acute proximal myopathy. Moreover, this patient presented solely with myopathy, without any of the classical symptoms or signs typically associated with hypoparathyroidism. It is highly unusual for multifactorial hypocalcemia to manifest exclusively as myopathy, making this case particularly rare and noteworthy.
